# Influence of Sevoflurane on the Neurological Pupil Index in Surgical and Critically Ill Patients: A Pilot Study

**DOI:** 10.3390/brainsci14030232

**Published:** 2024-02-28

**Authors:** Alice Dallemagne, Marco Anderloni, Mathias Havaux, Olivier Duranteau, Fabio Silvio Taccone

**Affiliations:** 1 Department of Anesthesia, Hôpital Universitaire de Bruxelles (HUB), Université Libre de Bruxelles (ULB), 1070 Brussels, Belgium; alicedallemagne@gmail.com (A.D.); mathias.havaux@hubruxelles.be (M.H.); olivier.duranteau@gmail.com (O.D.); 2 Department of Intensive Care, Hôpital Universitaire de Bruxelles (HUB), Université Libre de Bruxelles (ULB), 1070 Brussels, Belgium; marco.anderloni@studenti.univr.it

**Keywords:** neurological pupil index, inhaled sevoflurane, brain injuries, surgery, pupillary function

## Abstract

Background: The aim of this study was to compare the effects of sevoflurane on the neurologic pupil index (NPi), obtained by means of automated pupillometry, between intensive care unit (ICU) and surgical patients. Methods: This was a prospective single-center study conducted between December 2021 and February 2023. The eligible population comprised all patients undergoing general anesthesia (GA) for visceral surgery (VS) or neurosurgery (NS) and ICU patients receiving inhaled sevoflurane, according to the decision of the treating physician. The NPi measurements were conducted before GA (T0), after induction (T1), after the initiation of sevoflurane (T2), and at the point of discontinuation of sevoflurane (T3). Results: A total of 41 VS, 16 NS, and 22 ICU patients (out of which, 12 had a brain injury) were included. In the VS and NS groups, there was a significant decrease in the NPi over time, which remained within normal ranges. The NPi values decreased over time in the ICU group after sevoflurane administration. At T2, the NPi values were lower in the ICU group compared to the other groups. Lower NPi values were observed in the ICU patients with a brain injury compared to other patients. Conclusions: The administration of inhaled sevoflurane was associated with a significant reduction in the NPi values of the ICU patients with a concomitant brain injury.

## 1. Introduction

For several years, researchers have been striving to develop an effective tool for studying pupillary function. In particular, the pupil responds to light reaching the retina, with a signal being transmitted via the optic nerve to the Edinger–Westphal nucleus, which subsequently triggers the constriction of the pupil through the oculomotor nerve [1]. This pupillary light reflex (PLR) serves as a valuable indicator for assessing not only the retina and the presence of ocular diseases but also brainstem function [2]. Examining the brainstem is an integral part of the neurological evaluation of critically ill patients with cerebral lesions; the absence of pupillary reactivity is of prognostic significance, particularly after an anoxic injury [1,2,3]. In the field of anesthesiology, the PLR also holds significance as it can provide valuable information about the depth of anesthesia and analgesic effects. Pupillary constriction to light stimulation is reduced by propofol or inhaled anesthetics [4]. A reduction in pupillary size is also associated with the hypotension induced by general anesthesia [5].

However, the manual assessment of pupil size and PLR has some limitations that can affect its accuracy and reliability, including subjectivity, a lack of precision, and intra- or inter-observer variability [6]. To overcome these limitations, automated pupillometry has emerged as a valuable and easy-to-use alternative, offering objective and quantitative measurements of PLR [7]. Together with PLR, pupil size, constriction, and dilation velocity and latency in pupillary response can also be assessed, which result in more parameters to assess the pupillary response, providing valuable insights into underlying physiological processes and possibly being crucial for diagnosing certain pathological conditions [7]. All these parameters can be influenced by the administration of anesthetics or analgesics [8]; as such, automated pupillometry also provides the neurologic pupil index (NPi), which integrates all the available pupil-derived parameters into an ordinal scale from 0 (e.g., no pupillary response) to 5 (e.g., optimal pupillary response). The NPi is only marginally influenced by intravenous anesthetics and can be a reliable tool to assess PLR even in sedated or unconscious patients [8]. A lower NPi has been associated with severe post-anoxic brain injuries, increased intracranial pressure, or the occurrence of cerebral complications in patients undergoing extra-corporeal membrane oxygenation [3,9,10,11].

However, the NPi may also exhibit certain limitations. Apart from potential artifact or technical errors during data acquisition, the NPi can be influenced by pre-existing ocular diseases [12]. Additionally, severe hypercapnia has been associated with decreased NPi values, even in the absence of brain damage [13]. An intriguing case involved a patient resuscitated from cardiac arrest who exhibited an NPi value of 0 due to the use of sevoflurane, despite eventually experiencing neurological recovery and returning to normal NPi values upon their discontinuation of the drug [14]. These findings are noteworthy because, although sevoflurane is not commonly administered to patients in intensive care units (ICU), it is extensively employed in anesthesiology. In patients undergoing surgical procedures, the NPi has been shown to be significantly lower in those anesthetized with sevoflurane or desflurane compared to those receiving propofol within one hour of the surgery’s initiation, regardless of the depth of sedation [4]. Similar results have been observed in pediatric surgical patients anesthetized with sevoflurane [15]. These findings may suggest that inhaled anesthetics exert a more pronounced impact on midbrain reflexes than other drugs, although it remains unclear whether the presence of brain damage could further exacerbate these effects.

Therefore, the objective of this study was to compare the effects of inhaled sevoflurane on the NPi between ICU and surgical patients. Our hypothesis posited that patients with severe underlying conditions, such as those necessitating ICU admission, might exhibit more frequent pupillary abnormalities and lower NPi values when treated with inhaled sevoflurane.

## 2. Materials and Methods

### 2.1. Study Design

This was a prospective, comparative, and single-center study conducted at the Hôpital Universitaire de Bruxelles (HUB) between the 7 December 2021 and the 10 February 2023. This study received approval from the Ethical Committee of Erasme (REF B4062021000150—P2021/290), and written informed consent was obtained from eligible patients themselves or from their next of kin if the patients were unconscious at the time of inclusion, in accordance with local regulations. The eligible population comprised patients undergoing general anesthesia for visceral surgery (VS, including also gynecologic and urologic surgery) or neurosurgery (NS) as well as ICU patients receiving inhaled sevoflurane, as determined by the treating physician. Pupillary evaluation using automated pupillometry has been a routine procedure for ICU patients since 2020. The exclusion criteria for the patients in this study encompassed pre-existing ocular diseases and the absence of an NPi assessment during their hospitalization in the ICU. As this was an exploratory pilot study, no sample size calculation was performed. This study was reported according to the STROBE checklist for observational studies.

### 2.2. Data Collection

We collected demographic data alongside data regarding comorbid diseases (i.e., chronic kidney disease, heart failure, diabetes, chronic respiratory diseases) as well as the different drugs administered at the time of sevoflurane initiation. No further outcome data were collected.

### 2.3. Automated Pupillometry

Automated pupillometry was performed using the NeurOptics NPi-200 instrument (Neuroptics, Irvine, CA, USA). This device utilizes an infrared camera and delivers a standardized light stimulation with fixed intensity (1000 Lux) and duration (3.2 s). It enables a rapid and accurate measurement with a limit of 0.05 mm for pupil size as well as a quantitative assessment of the NPi and PLR, including the measurement of constriction velocity, latency, and the difference between the baseline and the post-stimulation pupil size expressed as a percentage of constriction from the baseline value. Abnormal NPi values were defined as below [3].

### 2.4. Study Protocol

The measurements were conducted in the operating room by the responsible anesthesiologist at four specific time points: T0, before the induction of general anesthesia (GA), with the patient in a supine position; T1, after the induction of GA, using a combination of propofol, sufentanil, and rocuronium; T2, after the initiation of inhaled sevoflurane at the target minimum alveolar concentration (MAC) level of 0.7–1; and T3, once the surgical procedure was completed and sevoflurane was discontinued (e.g., a minimum of MAC <0.3 was accepted for the final measurement). Inhaled sevoflurane was delivered through a vaporizer incorporated into the ventilator circuit.

In the ICU patients, who were already on anesthetic drugs at the moment in which the use of inhaled sevoflurane was decided, the NPi was measured (T1) before and 2 h after the initiation of therapy (T2); subsequent measurements were taken every 2 h, according to a local protocol; the target MAC were similar to the GA. Whenever available, the NPi upon the discontinuation of sevoflurane (T3) was also taken in these patients. Inhaled sevoflurane was delivered through the AnaConDa system (Sedana Medical, Danderyd, Sweden).

Each eye was evaluated separately, with the measurement process taking less than 30 s per eye and the contralateral eye being closed. The mean value of the two eyes was considered for this study.

### 2.5. Study Outcomes

The primary outcome of this study was the time-course of the NPi in different groups of patients (VS vs. NS vs. ICU). The secondary outcomes included the following: (a) the proportion of abnormal NPis at each time point among different groups; (b) the differences in the NPi values between the ICU patients with and without underlying brain damage (BD); and (c) the differences in pupil size and constriction rate (CH) over time and among different groups.

### 2.6. Statistical Analysis

The data in this article are expressed as a median (25th–75th percentiles) or a count (percentage). Comparisons between the VS, NS, and ICU patients for all the variables were performed using a Wilcoxon’s rank test for continuous variables or a Chi-square test, accordingly. Changes in repeated measurements within a group were assessed using Friedman’s test, with Dunn’s correction for multiple tests. Differences in repeated measurements among groups were assessed using a generalized mixed model (GMM), with Tukey’s post hoc correction; when the three groups were compared, only the T1, T2, and T3 time points were considered. The statistical analyses were performed using GraphPad PRISM version 5.0 (San Diego, CA, USA). For all the statistical tests, a *p* < 0.05 was considered significant.

## 3. Results

### 3.1. Study Population

**Figure 1:**
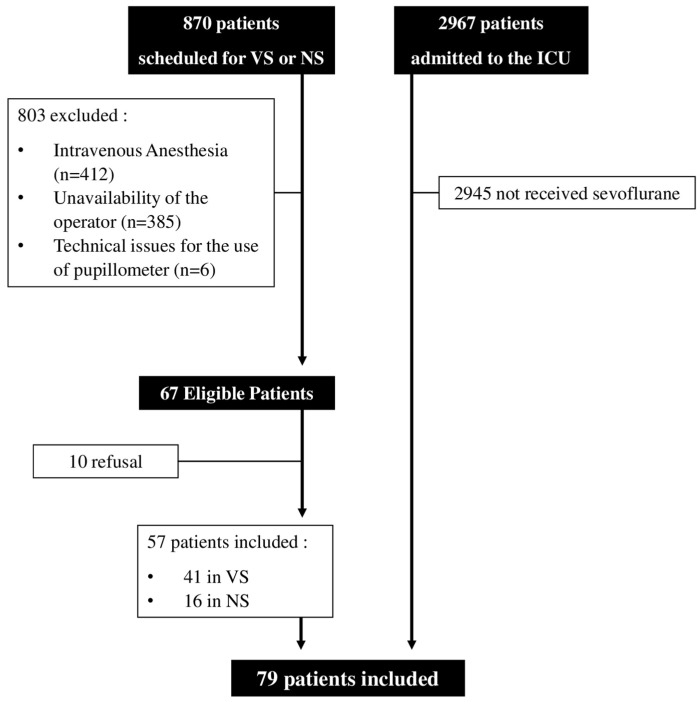
Flow-chart of this study. Intravenous anesthesia = only intravenous anesthetics were administered.

Over the study period, 870 patients were scheduled for elective VS or NS; of those, 57 (6.5%) patients were included (Figure 1), 41 of them after VS and 16 after NS.

Also, 2967 patients were admitted to the ICU; of those, 22 (0.1%) patients were treated with inhaled sevoflurane. The characteristics of the study population (n = 79) are reported in Table 1 and Supplementary Table S1.

The main indications for VS were the following: sleeve gastrectomy for obesity (n = 8); cholecystectomy (n = 7, all for uncomplicated gallbladder stones); gastrointestinal bypass for obesity (n = 6); gynecological surgery (n = 6, hysterectomy in five and endometrial ablation in one patient); colectomy (n = 2, both for colic cancer); and other conditions (n = 12, inguinal hernia repair in six patients, appendicectomy in three, and peritoneal biopsy in two patients). Fourteen of these VS procedures were performed as a laparoscopy (e.g., using carbon dioxide pneumoperitoneum). The main indications for NS were the following: brain tumor (n = 14, meningioma in seven patients, low-grade glioma in four, and glioblastoma in three patients), hypophysial adenoma (n = 1), and aneurysm clipping (n = 1). Among the ICU patients, 12 had an underlying BD (hemorrhagic stroke, n = 9; post-anoxic brain injury, n = 3), and 10 had no cerebral disorder (acute respiratory distress syndrome, n = 9, four of which suffered from COVID-19-related pneumonia; multimodal anesthesia for severe sickle cell disease, n = 1).

### 3.2. NPi Measurements

**Figure 2:**
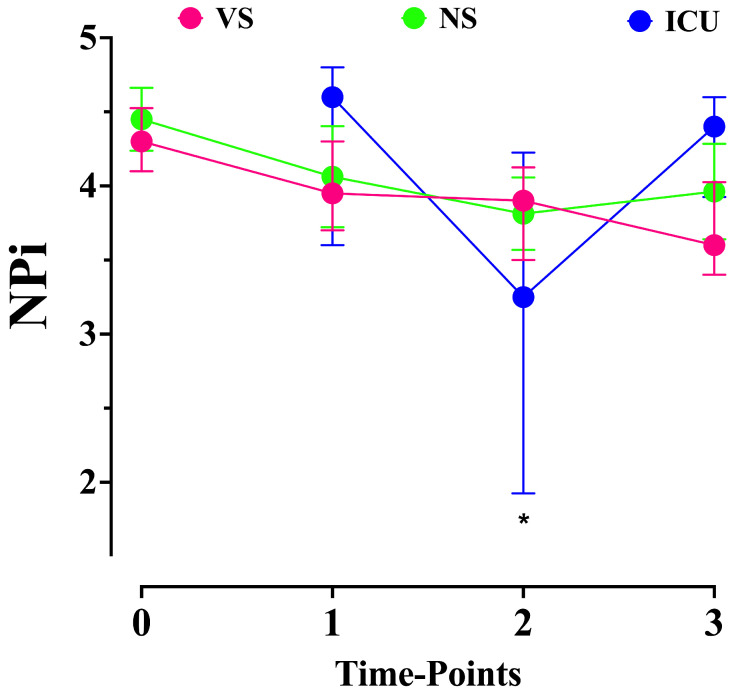
Neurologic pupil index (NPi) values over time in the three groups. * < 0.05 in the post hoc analysis for ICU vs. other groups (generalized mixed model for repeated measurements using only T1, T2, and T3; *p* < 0.001). VS = visceral surgery; NS = neurosurgery; and ICU = intensive care unit. 0 = T0; 1 = T1; 2 = T2; 3 = T3.

Data on the automated pupillometry assessments are shown in Table 2. The NPi measurements at T0 were similar between the VS and NS groups. Also, there was no significant difference in the NPi values among the three groups at T1 (Figure 2). There was a significant decrease in the NPi values over time in the VS group (*p* < 0.001); in particular, the T1, T2, and T3 measurements were significantly lower than the T0 values (Table 2); however, no difference between the NPi values before and after sevoflurane initiation (T1 vs. T2) was observed.

There was a significant decrease in the NPi values over time in the NS group (*p* < 0.001); in particular, the T1, T2, and T3 measurements were significantly lower than the T0 ones (Table 2); however, no difference between the NPi values before and after sevoflurane initiation (T1 vs. T2) was observed. There was a significant decrease in the NPi values over time in the ICU group (*p* < 0.001); in particular, the NPi values after sevoflurane initiation were significantly lower than those before drug administration and after its discontinuation. The NPi values over time were significantly different among the groups (*p* < 0.001); in particular, while the VS and NS groups had similar NPi measurements over time, the NPi values were significantly lower at T2 in the ICU group when compared with the VS and NS groups (Figure 2).

**Figure 3:**
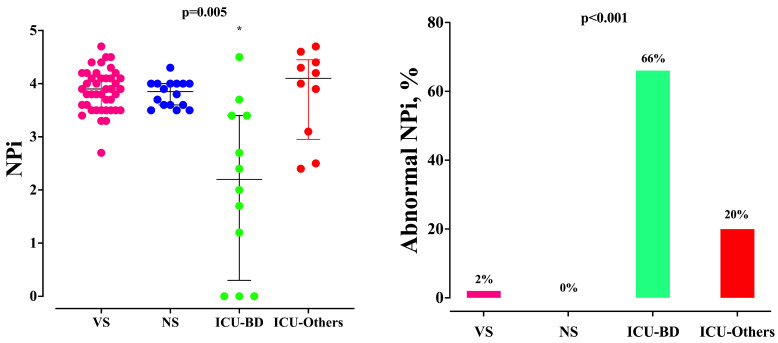
Neurologic pupil index (NPi) and the proportion of abnormal NPi at T2 between patients after visceral surgery (VS), neurosurgery (NS), or being admitted to the ICU with (BD) or without (“Others”) brain damage. * *p* < 0.05 vs. others.

The proportion of measurements with an abnormal NPi increased significantly over time only in the ICU group, in particular at T2 (Table 2). Moreover, the ICU patients with BD had lower NPi values at T2 than the others (Figure 3); also, the ICU patients with BD had an abnormal NPi after sevoflurane initiation more frequently than the others.

### 3.3. Other Measurements

Together with the NPi, there was a significant decrease in pupil size and CH values over time in the VS, NS, and ICU groups (*p* < 0.001); in particular, the T1, T2, and T3 measurements were significantly lower than the T0 values in the VS and NS group (Table 2), while the pupil size and CH values after sevoflurane initiation were significantly lower than those before drug administration and after its discontinuation in the ICU group (Table 2).

## 4. Discussion

In this study, we have shown that the NPi were significantly influenced by the initiation of inhaled sevoflurane only in the ICU patients. In particular, the NPi values decreased in the ICU patients with underlying brain damage compared to the others. In the patients undergoing a scheduled GA, the NPi decreased after sedation induction but was not further influenced by the administration of inhaled anesthetics. These findings suggest a relevant role of sevoflurane therapy on NPi assessment only in patients with underlying brain damage, in whom the use of such parameters to monitor brainstem dysfunction could become less reliable.

The assessment of pupil function is the cornerstone of neuro-monitoring in both surgical and ICU patients [7]. However, pupil size and pupillary response to different stimuli (i.e., light and pain) can be significantly influenced by many factors, including environment factors (i.e., light, pain), patient characteristics (i.e., age, eye color, ocular diseases, medications), brain damage (i.e., increased intracranial pressure, brainstem injury), and therapies (i.e., anesthetics and analgesics) [1]. Because of all these confounders, the use of automated pupillometry has been implemented to provide a reliable quantification of pupillary reflexes. Drugs play an important role in modifying these reflexes. Opioids, the most widely studied group of drugs in this context, cause miosis though the central disinhibition of Edinger–Westphal (EW) neurons but do not impact the NPi [16]. Intravenous anesthetics, such as propofol, decrease the CH, independent of the baseline pupillary size [17], but not the NPi [8]. In this setting, inhaled anesthetics, such as sevoflurane, can induce pupil miosis due to the suppression of inhibitory influences over the EW nucleus [18]. Also, inhaled anesthetics have been associated with a lower NPi than propofol in patients undergoing scheduled breast or thyroid surgery, which could suggest different effects of inhaled anesthetics on the alteration of midbrain reflexes when compared to intravenous anesthesia, although the NPi values in the study in question remained within normal ranges [4]. However, in another study including pediatric surgery, the NPi values were similar under inhaled and intravenous anesthesia [15]. These findings may be attributed to the concentrations of inhaled sevoflurane or the concomitant administration of other anesthetic and analgesic drugs, which can potentially interfere with pupillary function. In our study, the NPi exhibited a significant decrease upon anesthesia induction; however, its subsequent changes were not influenced by the initiation of sevoflurane. Moreover, the NPi values remained within normal ranges in most of the patients. We did not observe any differences between visceral surgery and neurosurgery, indicating that the type of surgical procedure did not impact the observed outcomes. Therefore, the utilization of automated pupillometry remains pertinent in the context of planned surgical interventions, considering that reduced NPi levels are highly uncommon in the absence of brain injury. As such, NPi monitoring can serve as a valuable tool for the early detection of cerebral complications during surgical procedures.

In critically ill patients in the ICU, low NPi values have been associated with brainstem dysfunction and increased intracranial pressure and hold significant prognostic value [3,7]. However, we observed a significant decrease in the NPi levels upon the initiation of inhaled sevoflurane, particularly among those patients with pre-existing brain damage. These findings could be explained by the potential additive effects of inhaled anesthetics and the underlying brain pathology of patients, resulting in notable alterations in pupillary function. Consequently, the administration of inhaled anesthetics in such patients becomes a noteworthy confounding factor when interpreting NPi values and should be taken into consideration in clinical practice. However, it is important to consider that a decrease in NPi values could also be influenced by the interaction of sevoflurane with rocuronium and sufentanil on pupillary function [19,20]. The use of inhaled anesthetics in critically ill patients remains relatively rare [21], although it has been an area of interest in recent years because of several potential advantages, including its rapid onset and offset of action, minimal metabolism in the body, and low potential for hepatic and renal toxicity [22]. One potential benefit of using inhaled sevoflurane in this patient population is its bronchodilator effect, which can help improve oxygenation and decrease airway resistance, such as in severe asthma [23]. In mechanically ventilated patients, inhaled sedation may reduce the time to extubation and weaning and has been shown to reduce opioid and muscle relaxant consumption [24,25]. Moreover, in patients suffering from acute respiratory distress syndrome, the use of inhaled sevoflurane improved oxygenation and decreased the levels of a marker of epithelial injury and inflammation when compared to midazolam [26]. However, the use of inhaled anesthetics can also contribute to the development of hypercapnia in some cases, which occurs due to the dead space resulting from the delivery device, air pollution, requiring the implementation of a dedicated scavenging system, and, although less commonly, the occurrence of malignant hyperthermia and diabetes insipidus [22]. Together with altered NPi values, these findings indicate the potential benefits of utilizing inhaled sevoflurane in critically ill patients; however, the administration of sevoflurane should be tailored to each patient and carefully evaluated through a comprehensive assessment of the associated risks and benefits.

This study has several limitations that should be acknowledged. Firstly, it is important to note that this study was conducted as a pilot study, and, therefore, no sample size calculation was performed. To establish more robust conclusions, larger confirmatory studies with appropriate sample size calculations are necessary. Secondly, a specific evaluation of whether optimal conditions for a pupillometry assessment, such as ambient light, were consistently maintained during all measurements was not performed. This can be challenging to achieve in an operating room setting where lighting conditions may vary. Thirdly, it is worth mentioning that some patients in both the VS and NS groups still had residual sevoflurane expired concentrations at the time of the last measurement. This residual presence of sevoflurane could potentially account for the lower NPi values compared to the baseline measurements. Also, we did not record the length of surgery and the cumulative fentanyl doses received during the surgical procedures; however, these conditions were unlikely to influence the NPi measurements. Fourthly, the impact of other concurrent medications, such as opioids or intravenous anesthetics (e.g., ketamine), on NPi measurements was not specifically evaluated in this study. Also, we could not compare the effects of sevoflurane on the NPi in comparison with other techniques assessing depth of sedation or analgesia narcosis. It would have also been interesting to compare whether NPi changes could predict the speed of consciousness recovery after surgery; this should be assessed in future studies. Fifthly, it is essential to highlight that this study focused solely on sevoflurane and cannot provide conclusions regarding the effects of other inhaled anesthetics, such as isoflurane and desflurane. Considering these limitations, future studies addressing these concerns are warranted to provide a more comprehensive understanding of the effects and implications of inhaled anesthetics on NPi measurements in various clinical scenarios.

## 5. Conclusions

In this study, the administration of inhaled sevoflurane demonstrated a notably greater reduction in the NPi values among critically ill patients with brain injuries compared to surgical or other ICU populations. These findings suggest that the use of inhaled anesthetics may represent a significant confounding factor in the interpretation of NPi values specifically in this patient population.

## Figures and Tables

**Table 1:** Characteristics of the study population. Data are presented as a count (%) or a median (25th–75th percentiles). VS = visceral surgery; NS = neurosurgery; and ICU = intensive care unit.

	All (n = 79)	VS (n = 41)	NS (n = 16)	ICU (n = 22)	*p* Value
**Age, years**	50 (20–76)	48 (20–76)	60 (35–75)	52 (24–72)	<0.001
**Female gender, n (%)**	43 (54.5)	29 (70.7)	4 (25.0)	10 (45.5)	0.005
**Cardiovascular disease, n (%)**	36 (45.6)	15 (36.6)	9 (56.3)	12 (54.5)	0.25
**Diabetes, n (%)**	11 (13.9)	3 (7.3)	3 (18.8)	5 (22.7)	0.20
**COPD, n (%)**	10 (12.7)	5 (12.2)	2 (12.5)	3 (13.6)	0.99
**Glaucoma, n (%)**	2 (2.5)	0	1 (6.3)	1 (4.5)	0.48
**Previous Neurologic Disease, n (%)**	8 (10.1)	4 (9.8)	3 (18.8)	1 (4.5)	0.36

**Table 2:** Automated pupillometry assessment in the study population. Data are presented as a count (%) or a median (25th–75th percentiles). VS = visceral surgery; NS = neurosurgery; ICU = intensive care unit; NPi = neurologic pupil index; and CH = constriction rate.

		T0	T1	T2	T3	*p* Value
**NPi**	VS, n = 41	4.3 (4.1–4.5)	3.9 (3.7–4.2)	3.9 (3.5–4.2)	3.7 (3.4–4)	<0.001
	NS, n = 16	4.5 (4.3–4.6)	4.0 (3.8–4.4)	3.8 (3.5–4)	4.0 (3.7–4.2)	<0.001
	ICU, n = 22	-	4.6 (3.6–4.8)	3.8 (2.5–4.3)	4.4 (3.9–4.6)	<0.001
**Pupil Size**	VS, n = 41	4.1 (3.6–4.6)	2.9 (1.9–2.6)	1.9 (1.7–2.2)	2.2 (1.8–2.6)	<0.001
	NS, n = 16	3.6 (3.3–4)	2.0 (1.9–2.2)	1.7 (1.5–2)	2.0 (1.8–2.3)	0.001
	ICU, n = 22	-	3.4 (2.8–4.7)	3.2 (2.4–4.6)	3.1 (2.6–4.3)	0.01
**CH, %**	VS, n = 41	33 (28–38)	9 (6–14)	7 (5–11)	7 (4–11)	<0.001
	NS, n = 16	29 (24–35)	9 (6–13)	7 (4–9)	9 (6–12)	<0.001
	ICU, n = 22	-	31 (25–37)	17 (10–27)	27 (20–36)	<0.001
**Abnormal NPi**	VS, n = 41	0	1 (2)	1 (2)	3 (7)	0.27
	NS, n = 16	0	0	0	0	-
	ICU, n = 22	-	3 (13.6)	10 (62.5)	4 (25)	0.03

## Data Availability

The datasets used and/or analyzed during the current study are available from the corresponding author upon reasonable request. Due to we have ethics restrictions for data sharing.

## Supplementary Materials

The following supporting information can be downloaded at https://www.mdpi.com/article/10.3390/brainsci14030232/s1: Table S1: Characteristics of the ICU population.
