# Supplementary material for: Influence of Sevoflurane on the Neurological Pupil Index in Surgical and Critically Ill Patients: A Pilot Study
Source: Brain Sci. 2024 Feb 28;14(3):232. doi: 10.3390/brainsci14030232 (PMC10968887; doi:10.3390/brainsci14030232)
Supplement: Supplementary file 1 [file brainsci-14-00232-s001.zip › brainsci-2870074-supplementary.pdf]

Supplemental Table S1: Characteristics of the ICU population.

	N=22
**Male Gender – n (%)**	12 (54.5)
**Age – years**	52 (24-72)
**Comorbidities– n (%)**	
Cardiovascular diseases	12 (54.5)
Diabetes	5 (22.7)
COPD	3 (13.6)
Glaucoma	1 (4.5)
Previous neurologic Disease	1 (4.5)
Alcohol consumption	6 (27.3)
**Drugs on the day of sevoflurane – n (%)**	
Ketamine	2 (9.1)
Sufentanil	13 (59.1)
Morphine	1 (4.5)
Propofol	22 (100.0)
**Length of ICU stay, days**	26 (6-149)
**ICU Survival, n (%)**	14 (63.6)

Characteristics of the VS population.

	N=41
**Male Gender – n (%)**	12 (29)
**Age – years**	48 (20-76)
**Comorbidities– n (%)**	
Cardiovascular diseases	15 (36.5)
Diabetes	3 (7.3)
COPD	5 (12.2)
Glaucoma	0
Previous neurologic Disease	4 (9.8)
**Drugs during anaesthesia – n (%)**	
Ketamine	14 (34)
Amines	3 (7.3)
Clonidine	16 (39)

Characteristics of the NS population.

	N=16
**Male Gender – n (%)**	12 (75)
**Age – years**	60 (35-75)
**Comorbidities– n (%)**	
Cardiovascular diseases	9 (56.4)
Diabetes	3 (18.8)
COPD	2 (12.5)
Glaucoma	1 (6.3)
Previous neurologic Disease	3 (18.8)
**Drugs during anaesthesia – n (%)**	
Ketamine	0 (0)
Amines	0 (0)
Clonidine	12 (75)
